# Periosteal Surgery in the United States

**Published:** 1877-08

**Authors:** 


### ARTICLE VII.

### *Periosteal Surgery in the United States.*

In a short editorial with the above heading *The Lancet* (June 2, 1877,) says: "We have been favoured with a look at the New Lower Jaw-bone alluded to in our report of the Congress of German surgeons at Berlin, and which we believe to be, if not a unique specimen at any rate the first specimen of the sort seen in Europe. We allude to it with the more pleasure, as the operator, Dr. James R. Wood, Emeritus Professor of Surgery, in the Bellevue Hospital Medical College, is entitled to the great praise of having been one of the pioneers of periosteal surgery, which constitutes such a creditable and instructive chapter in the recent history of surgery. This particular operation was performed more than twenty years ago; and the merit of it consists of not only in its having been then a new kind of operation, but in the details of the procedure, which had to be thought out for the first time, and which have since become recognized principles.

"It is a great feat of what we are disposed to call physiological surgery to take away a whole bone, and to do it so carefully and with such preservation of the periosteum as to have it entirely reproduced in perfect symmetry and perfectly *in situ*. The new Jaw is smaller than the original one, but in other respects, in form and position, it is a wonderfully perfect reproduction. The patient was a girl eighteen years of age, working in a match factory; hence the phosphorous disease of the jaw, leading to necrosis and the necessity for removal. The operation was done by halves, one-half being left for weeks after the removal of the other, so as to steady the parts and determine the proper position of the new jaw, which would otherwise have been dragged down by muscles and caused great deformity. The patient perfectly recovered, and lived three years after. She then died of brain abscess, when the entire skull came into the possession of Dr. Wood. Both he and other operators have frequently repeated these operations with similar success. But patients are mostly alive, and, as Langenback lately said at Berlin, there is not another such specimen in the whole of Europe as the one we now notice.—*Med. News and Library.*

